# Comprehensive infection control measures prevent hospital-acquired severe acute respiratory syndrome coronavirus 2 infection: A single-center prospective cohort study and seroprevalence survey

**DOI:** 10.1371/journal.pone.0257513

**Published:** 2021-10-11

**Authors:** Hiroshi Hori, Takahiko Fukuchi, Masamitsu Sanui, Takashi Moriya, Hitoshi Sugawara

**Affiliations:** 1 Division of General Medicine, Department of Comprehensive Medicine 1, Saitama Medical Center, Jichi Medical University, Saitama City, Japan; 2 Division of Anesthesiology and Critical Care Medicine, Department of Comprehensive Medicine 2, Saitama Medical Center, Jichi Medical University, Saitama City, Japan; 3 Division of Emergency and Critical Care Medicine, Department of Comprehensive Medicine 1, Saitama Medical Center, Jichi Medical University, Saitama City, Japan; Waseda University: Waseda Daigaku, JAPAN

## Abstract

**Background:**

Coronavirus disease (COVID-19) is associated with a high mortality rate in older adults; therefore, it is important for medical institutions to take measures to prevent severe acute respiratory syndrome coronavirus 2 (SARS-CoV-2) transmission. This study aimed to assess the risk of SARS-CoV-2 infection among healthcare workers (HCWs) and the effectiveness of infection control measures.

**Methods:**

This study had a cross-sectional component and a prospective cohort component. The cross-sectional component comprised an anti-SARS-CoV-2 antibody survey among HCWs at a medical center in Saitama City, Japan. In the prospective cohort component, HCWs at the same medical center were tested for anti-SARS-CoV-2 antibodies monthly over a 3-month period (May to July 2020) to assess the effectiveness of infection prevention measures, including personal protective equipment use. All participants in the cohort study also participated in the antibody survey. The primary outcome was anti-SARS-CoV-2 antibody (measured using Elecsys^®^ Anti-SARS-CoV-2) positivity based on whether participants were engaged in COVID-19-related medical care. Other risk factors considered included occupational category, age, and sex.

**Results:**

In total, 607 HCWs participated in the antibody survey and 116 doctors and nurses participated in the cohort study. Only one of the 607 participants in the survey tested positive for anti-SARS-CoV-2 antibodies. All participants in the cohort study were anti-SARS-CoV-2 antibody negative at baseline and remained antibody negative. Engaging in the care of COVID-19 patients did not increase the risk of antibody positivity. During the study period, a total of 30 COVID-19 in-patients were treated in the hospital.

**Conclusions:**

The infection control measures in the hospital protected HCWs from nosocomially acquired SARS-CoV-2 infection; thus, HCWs should engage in COVID-19-related medical care with confidence provided that they adhere to infectious disease precautions.

## Introduction

As of June 5, 2021, there have been over 172 million cases of COVID-19 and over 3.71 million deaths (https://coronavirus.jhu.edu/map.html).

Because the COVID-19-related morbidity and mortality are higher in persons aged ≥70 years than in younger persons [1, 2], a high number of deaths of older adults have occurred in clusters in healthcare facilities [3]. Approximately 50% of COVID-19 clusters that have occurred in Japan have been related to healthcare and long-term care facilities [4]. Japan has many hospitals and the oldest population worldwide [5]. Therefore, SARS-CoV-2 infection prevention measures in healthcare institutions are extremely important.

Several studies have shown that healthcare workers (HCWs) have a higher anti-SARS-CoV-2 antibody seroprevalence than the general population, which indicates a risk of transmission in medical care settings [6–8]. There are many mild or asymptomatic cases of SARS-CoV-2 infection, and thus, it is possible for asymptomatic HCWs to transmit the infection to older patients [9]. To prevent nosocomial transmission, it is extremely important to prevent infection among HCWs; however, there is limited information on the effectiveness of in-hospital infection prevention measures.

Saitama Prefecture, where the hospital is located, has the fourth largest cumulative number of COVID-19 cases in Japan and is adjacent to Tokyo, which has the largest cumulative number of cases. Since April 2020, the hospital has been treating patients with moderate and severe COVID-19.

Therefore, in this study, we measured the anti-SARS-CoV-2 antibody in hospital staff to evaluate the risk of infection among HCWs and the effectiveness of infection control measures (cross-sectional study). As antibodies are undetectable in the early stage of infection and may become negative over time after infection [10, 11], we also conducted a prospective cohort study in which we measured the antibody positivity at three time points to determine the risk of acquiring infection in a COVID-19-related medical care setting.

## Methods

### Study design and participant recruitment

The participants were HCWs who had been working at the medical center since April 2020. The participants in the cohort study were physicians and nurses working at the critical care center, COVID-19 ward, and the intensive care unit and who had many opportunities to directly engage in the treatment of patients with COVID-19. Participants in the cohort study were limited to doctors and nurses because this cohort focused on HCWs who had direct contact with patients with COVID-19, which is associated with a high risk of infection. From May 2020 to July 2020, doctors and nurses were the only HCWs in direct contact with COVID-19 patients, who worked full time in the COVID-19 ward, and could continue to participate in our study.

The cross-sectional study included physicians, nurses, nursing assistants, therapists, midwives, pharmacists, radiologic technologists, clinical technologists, and clinical engineers. This study included occupations other than doctors and nurses to assess the risk of infection according to occupation.

**Fig 1:**
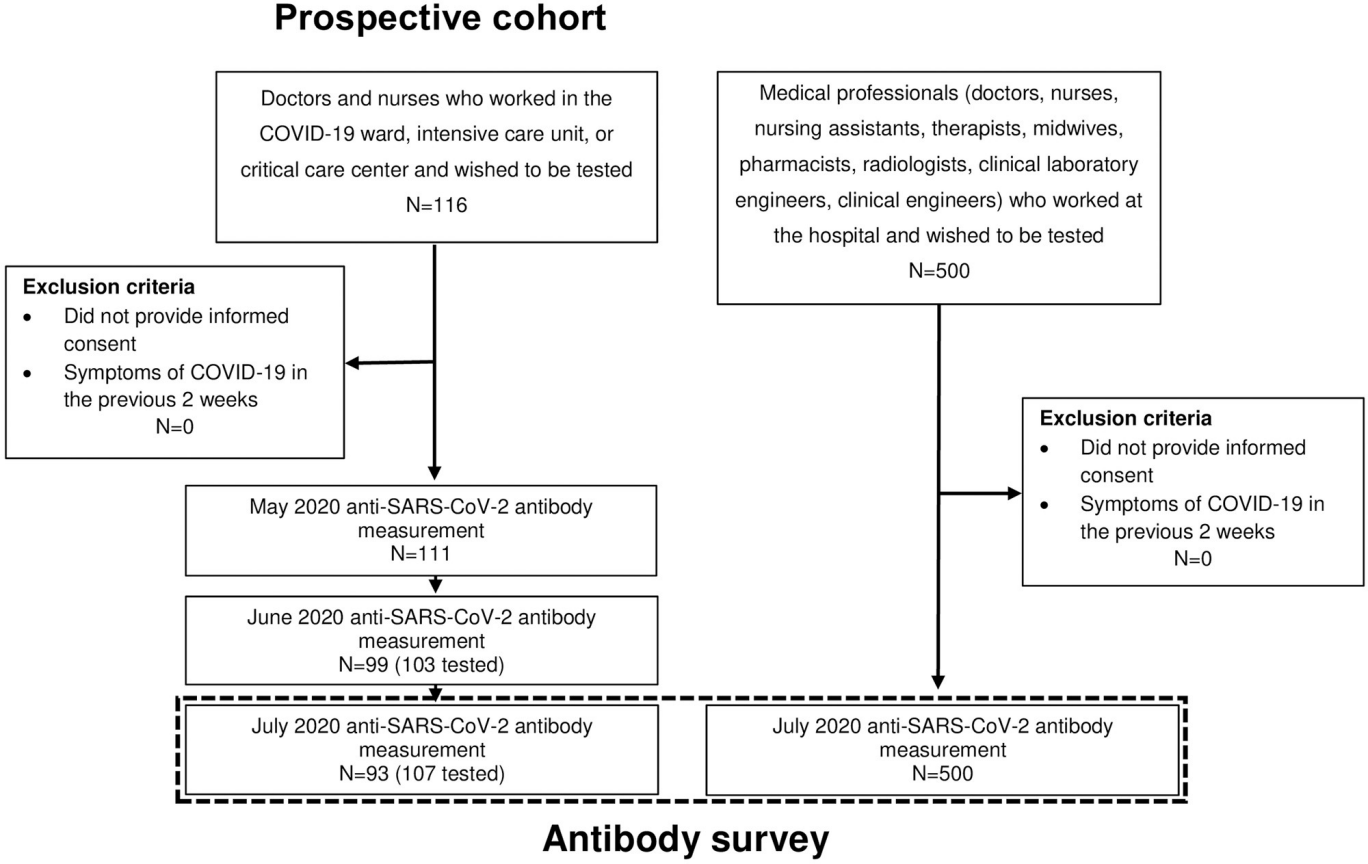
Flow chart: Recruitment for the prospective cohort study and anti-SARS-CoV-2 antibodies’ survey among healthcare workers. In the cohort study, the N in May, June, and July is the number of participants who were tested consecutively from the previous month, and the number in parentheses is the total number of participants who were tested in that month.

All participants in the cohort study also participated in the cross-sectional study (Fig 1). Participants were recruited to the prospective cohort study and antibody survey from April 27, 2020 and June 26, 2020, respectively. All participants were followed up until December 2, 2020.

The exclusion criteria were symptoms of possible SARS-CoV-2 infection (fever, rhinitis, cough, sore throat, respiratory distress, dysgeusia) during the previous 2 weeks and lack of consent. Participants were recruited through posters publicizing the study and e-mails. All eligible HCWs who agreed to participate were enrolled.

### Infection prevention measures

The infection control measures are shown in Table 1. Throughout the study period, the infection control team provided HCWs with regular guidance on infection countermeasures. All HCWs were instructed to wear masks, ensure thorough hand hygiene, and refrain from eating meals with many people inside the hospital.

**Table 1:** Comprehensive infection control measures to prevent COVID-19 in Saitama Medical Center.

Target	Nonpharmaceutical interventions to prevent COVID-19
COVID-19^a^ specialized ward, EICU^a^ dedicated to critically ill people	• Establishing a dedicated COVID-19 ward and EICU dedicated to patients with severe symptoms
	• Exclusive duties for healthcare practitioners in the dedicated ward
	• Zoning in the dedicated ward
	• Wearing of personal protective equipment in the dedicated ward
	• Course on wearing and removing personal protective equipment
	• Mental health intervention in medical care
Guidance to all hospital staff	• Ensuring wearing of masks within the hospital and during commute
	• Ensuring social distancing
	• Notification to refrain from eating meals with a large number of persons
	• Stopping face-to-face seating in the hospital cafeteria
	• Avoiding situations with a high infection risk (e.g., crowded places, close-contact settings, confined and enclosed spaces)
	• Thorough guidance regarding hand hygiene
	• Regular COVID-19 workshops
	• Mental health intervention for hospital staff
Notices	• Ensuring ventilation
	• Prohibition of hospital visits
	• Check fever and symptoms of all patients and hospital staff at the entrance
Fever support in the emergency outpatient department and general outpatient departments	• Ward selection after admission and precaution selection by utilizing the predicted score of COVID-19 using chest imaging, blood sampling, and interviews in emergency outpatient and general outpatient hospitalization.
	• Change in response in accordance with the outbreak status

^a^COVID-19, coronavirus disease; EICU, emergency intensive care unit.

Moreover, hospital staff members were provided with guidance on thorough basic infection prevention measures for daily life outside of the hospital setting. In particular, staff members were educated on wearing masks outside their home; ensuring thorough hand hygiene; and avoiding unnecessary and nonurgent outings, situations with a high risk of infection (e.g., crowded places, close-contact settings, confined and enclosed spaces), and eating meals with many people.

### Compliance with infection prevention measures

In both the cohort study and antibody survey, a structured questionnaire was administered whenever blood was drawn for antibody testing, to assess adherence to infection prevention measures, including thorough hand hygiene and avoidance of unnecessary and nonurgent outings and situations with a high risk of contracting the infection.

In addition, the amount of hand hygiene detergent used in the hospital was measured and compared with the amount used before the infection prevention measures were implemented.

### Anti-SARS-CoV-2 antibody testing

Based on the results of antibody testing conducted in the Tokyo metropolitan area [12], the antibody-positive rate was predicted to be ≤1%. Therefore, it is important to use highly specific antibodies to reduce the false-positive rate [13]. In a systematic review of the diagnostic accuracy of antibody tests, the specificity of enzyme-linked immunosorbent assay and chemiluminescent immunoassay was reported to be high [14]; therefore, we used Elecsys^®^ Anti-SARS-CoV-2 (Roche Diagnostics), a chemiluminescent immunoassay. According to the manufacturer, the antibody test has a sensitivity of 100% (95% confidence interval [CI]: 88.1–100%) and specificity of 99.81% (95% CI: 99.65–99.91%) [15].

To confirm the sensitivity, patients who were admitted to the hospital with COVID-19 confirmed using a positive SARS-CoV-2 PCR test result were tested for antibodies (both Elecsys^®^ Anti-SARS-CoV-2 and Architect SARS-CoV-2 IgG [Abbott Laboratories Inc.]). All 10 patients with COVID-19 tested positive for anti-SARS-CoV-2 antibodies on both tests.

Additionally, the samples collected in May 2020 were tested for anti-SARS-CoV-2 IgG using Architect SARS-CoV-2 IgG [16] to check the consistency of the results. Any positive antibody results were confirmed using the Architect SARS-CoV-2 IgG Assay and Cica Immuno-test SARS-CoV-2 IgG [17].

### Data collection

#### Prospective cohort study

In the prospective cohort study, blood samples were collected from participants in May 2020 and 1 and 2 months later, and the samples were tested for anti-SARS-CoV-2 antibodies. At the start of the study, information was collected on age, sex, municipality of residence, method of commuting, underlying illnesses, medication history, smoking history, work department, BCG history, and travel history during the past year, using a structured questionnaire. At each study visit, the following information was recorded: number of COVID-19 patients under the participant’s care in the past month, contact with a COVID-19 cluster site, possible symptoms of COVID-19, and COVID-19 diagnosis during the past month.

#### Antibody survey

In the antibody survey, blood samples were collected for antibody testing during a 2-week period in July 2020. At the time of blood sample collection, information was collected on age, sex, profession, work department, history of providing care for patients with confirmed COVID-19 and the timing, municipality of residence, means of commuting (i.e., use public transportation such as buses or trains), underlying illness, smoking history, BCG history, travel history during the past year, and presence of possible symptoms of COVID-19 in the past 4 months.

### Outcomes and risk factors

In both the prospective cohort study and the survey, the primary outcome was anti-SARS-CoV-2 antibody positivity. Risk factors for antibody positivity were also assessed, including sex, age (≥40 years), use of public transportation for commuting, municipality of residence (Saitama City, Tokyo Metropolis, or other), underlying illness, smoking history, BCG vaccination history, work in the emergency unit, performing endoscopic examinations, and the presence of possible symptoms of COVID-19 in the past 4 months.

### Sample size

Based on the results of antibody testing conducted in Tokyo [10], we calculated that the sample size required was 381 people, assuming a prevalence of 1% and a margin of error of 1% in a cross-sectional study.

### Statistical analysis

We summarized participant characteristics using proportions (sex and occupation of HCWs) or medians and interquartile ranges (age). The prevalence of anti-SARS-CoV-2 antibodies was calculated with the corresponding 95% CI. For descriptive statistics, we determined the differences between the anti-SARS-CoV-2 antibody-positive and -negative groups using Fisher’s exact test for categorical variables and the Mann–Whitney *U* test for continuous variables in both the prospective cohort study and the survey. We performed multivariable logistic regression to assess risk factors for antibody positivity. P-values < 0.05 were considered statistically significant. Microsoft Excel 2013 version 15.0.5319.1000 (Microsoft Corp., Redmond, WA) and R version 3.6.0 (R Foundation for Statistical Computing, Vienna, Austria) were used for data analysis.

### Ethics approval and consent to participate

The Clinical Research Ethics Review Board of Saitama Medical Center, Jichi Medical University, Japan, approved this study (Clinical S20-011 [cohort study], Clinical S20-046 [antibody survey]). All participants provided written informed consent.

## Results

Table 2 shows the characteristics of participants in the antibody survey and the cohort study. There were 116 participants in the cohort study, with a median age of 29 years, of whom 74.2% were female. Of the 116 participants in the cohort study, 111, 103, and 107 underwent blood sampling in May, June, and July, respectively. A total of 93 participants participated in all three blood sample collections (Fig 1). There were 607 participants in the cross-sectional study, including 107 who participated in the cohort study, with a median age was 34 years, of whom 70.1% were female. The proportion of participants engaged in COVID-19-related medical care was 75.9% and 26.7% in the cohort study and the survey, respectively.

**Table 2:** Participant characteristics.

	Survey	Cohort study
Participants	607^†^	116
Age: median (interquartile range) (years)	34 (27–41)	29 (25–37)
Sex: male (%)	188 (30.9%)	30 (25.8%)
Occupation		
Doctor	173	38
Nurse, nursing assistant	289	78
Midwife	16	0
Pharmacist	14	0
Radiologist	29	0
Clinical engineer	12	0
Clinical laboratory engineer	54	0
Therapist	13	0
Nutritionist	7	0

^†^ This total of 607 includes all participants in the cohort study.

All participants in the cohort study tested negative for antibodies in the first, second, and third tests (Table 3). The samples collected in May 2020 were also tested using Architect SARS-CoV-2 IgG (Abbott), and all results were negative.

**Table 3:** Anti-SARS-CoV-2 antibody test results of the prospective cohort study.

Timing of the antibody test	Number of participants tested	Number of continuing participants	Number of participants showing anti-SARS-CoV-2 antibody positivity
May 2020	111	111	0 (0.0%)
June 2020	103	99	0 (0.0%)
July 2020	107	93	0 (0.0%)

In the antibody survey, only one participant tested positive for anti-SARS-CoV-2 antibody, with a seroprevalence of 0.16% (95% CI: 0.008–1.06%) (Table 4). On confirmatory testing, the Architect SARS-CoV-2 IgG result was negative, and the Cica Immuno-test SARS-CoV-2 IgG result was positive; thus, the results were discordant. The antibody-positive participant was a nurse who did not engage in COVID-19-related medical care and had not experienced symptoms within the past 4 months and was, therefore, assumed to have experienced asymptomatic infection.

**Table 4:** Results of the antibody survey.

		Anti-SARS-CoV-2 antibody test			
	Number	Positive	Negative	Antibody prevalence (%)	P value^†^
All participants	607	1	606	0.165 (95% CI: 0.008–1.060)	
Age (over 40 years)	168 (27.8%)	0	168	0.000	>0.99
Sex (male)	188 (30.9%)	0	188	0.000	>0.99
Engagement in COVID-19 medical care	162 (26.7%)	0	162	0.000	>0.99
Doctors	173 (28.5%)	0	173	0.000	>0.99
Nurses, nursing assistants	289 (47.6%)	1	288	0.347	0.476
Other than doctor or nurse	145 (23.9%)	0	145	0.000	>0.99
Uses public transportation for commuting	189 (31.5%)	0	189	0.000	>0.99
Lives in Saitama City	458 (76.3%)	1	457	0.002	>0.99
Lives in Tokyo City	37 (6.2%)	0	37	0.000	>0.99
Lives in another city	105 (17.5%)	0	105	0.000	>0.99
Symptoms within 4 months	132 (21.7%)	0	132	0.000	>0.99
Underlying disease	94 (15.8%)	0	94	0.000	>0.99
Smoking	57 (9.5%)	0	57	0.000	>0.99
BCG inoculation	499 (94.7%)	1	498	0.002	>0.99
Engaged in the critical care center	84 (13.9%)	0	84	0.000	>0.99
Engaged in endoscopy	23 (3.8%)	0	23	0.000	>0.99

^†^ Fisher’s exact test.

Multivariate analysis showed no difference in the antibody-positive rate between persons who engaged in COVID-19-related medical care and those who did not in the cross-sectional study. In the cross-sectional study, no significant differences were noted in antibody seroprevalence according to smoking history, occupation, symptoms suggestive of COVID-19 within the previous 4 months, travel history, municipality of residence, or use of public transportation to commute (Table 4).

**Fig 2:**
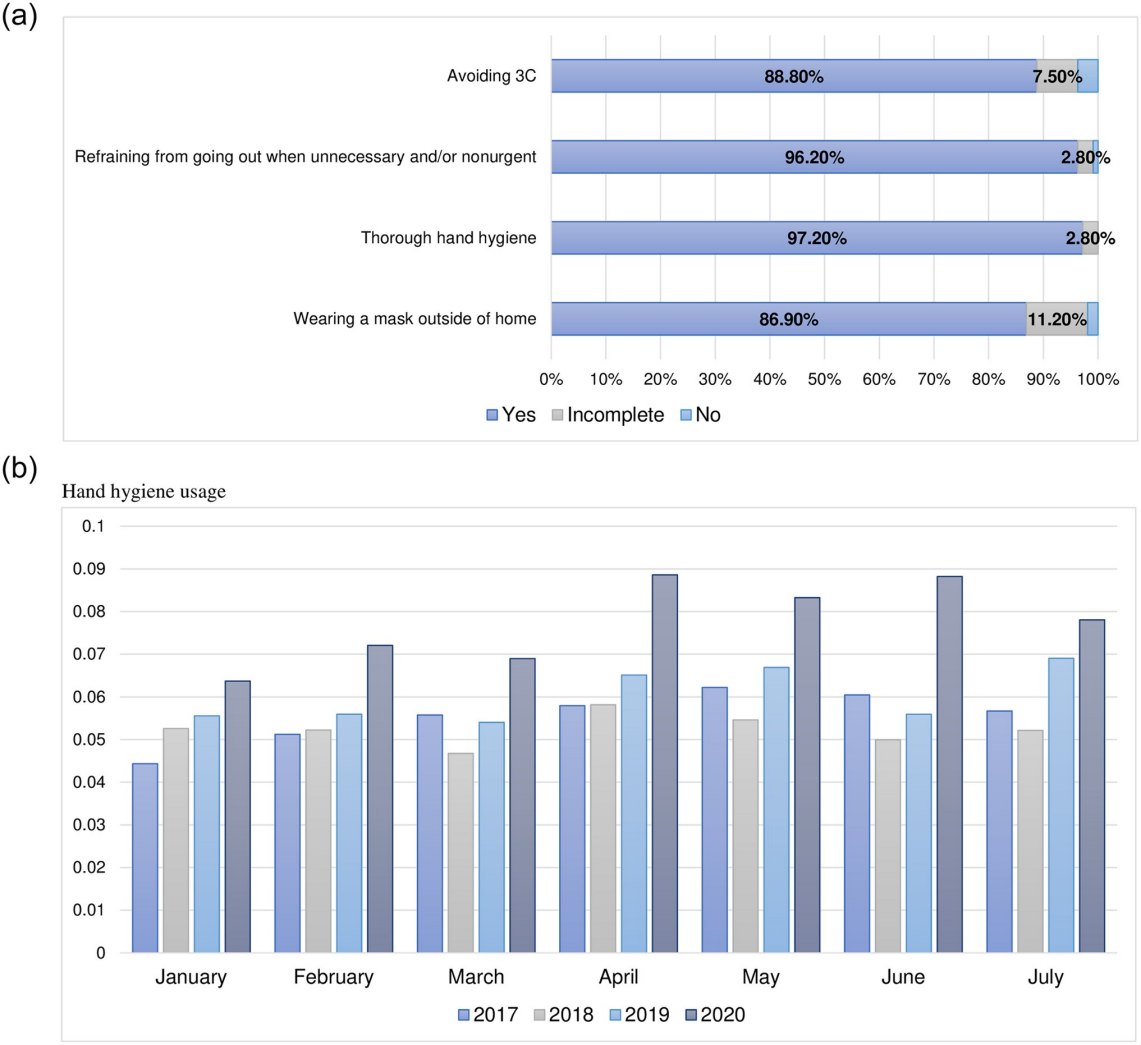
Use of prevention measures. a. Rate of compliance with infection prevention measures. b. Hand hygiene product usage according to month and year at the study hospital. The monthly hand hygiene product usage was calculated by dividing the usage of hand sanitizer used by total number of days for hospital patients in the month. Hand hygiene product usage was higher in 2020 than in the three preceding years for all the months studied. *3Cs: Crowded places, Close-contact settings, Confined and enclosed spaces.

In the cohort study, compliance with infection prevention measures, including thorough hand hygiene and avoidance of unnecessary and nonurgent outings and situations with a high risk of infection, was reported to be very high (Fig 2a). Compared with the previous year, total consumption per patient-hospital-day increased in 2020 (Fig 2b).

During the study period, a total of 30 COVID-19 in-patients were treated in the hospital. Most were moderately or severely ill patients requiring oxygen administration, mechanical ventilation, or extracorporeal membranous oxygenation.

## Discussion

At the time of the cross-sectional study in July 2020, the seroprevalence of anti-SARS-CoV-2 antibodies in the 607 HCWs tested was 0.16%, and there was no difference based on engagement in COVID-19-related medical care. Furthermore, from May to July 2020, antibody test results of physicians and nurses engaged in COVID-19-related medical care were all negative, and there was no evidence of nosocomial transmission.

In this study, there was no difference in the seroprevalence of anti-SARS-CoV-2 antibodies according to the level of engagement in COVID-19-related medical care in either the survey or cohort study. As there was an extremely high compliance with infection control measures, these results suggest that such measures may be effective in preventing infection outside the hospital environment. High consumption of hand hygiene products confirmed that hand hygiene was being performed more actively in this hospital, which was likely to be an important factor for preventing infection among HCWs.

### Effectiveness of previous reports on the usefulness of specific infection prevention measures

HCWs are at a particularly higher risk of infection than the general population [18–20]. Moreover, inadequate hand hygiene before and after contact with a patient, long working hours, and improper use of personal protective equipment (PPE) increases the risk of infection. Thorough hand hygiene and appropriate PPE use and mask-wearing are particularly important [20–22]. A systematic review of PPE use and exposure to contaminated body fluids of infected patients reported that the procedures for wearing and removing PPE, i.e., removing the gown according to the Centers for Disease Control and Prevention removal guidance, using double gloves, and disinfecting gloves when undressing, reduce contamination and improve compliance [23].

In the hospital, double gloves and a gown covering the forearms to prevent a gap between the glove and the gown were used as PPE. Moreover, guidance on donning and doffing PPE was provided regularly, and thorough training was conducted. Among HCWs, this might have played an important role in preventing infection.

The study results support the use of PPE in COVID-19 patient care, and HCWs can significantly reduce the risk of infection if adequate infection control measures are taken. Liu et al. [24] also reported that anti-SARS-CoV-2 antibodies were not detected in 420 HCWs and that appropriate infection control measures such as PPE use prevented the infection. Comprehensive infection prevention measures give HCWs engaged in COVID-19 medical care confidence.

### Strengths and limitations

A strength of this study is that to the best of our knowledge, this is the first study in which antibodies were repeatedly measured in the same population. Serial measurements make it possible to solve the problem of negative antibody results over time and false-negative results in early infection because of the window period before the result becomes positive. Furthermore, blood samples from 10 hospital in-patients with confirmed COVID-19, collected 7 days after the onset of their symptoms, tested positive, confirming the sensitivity of the antibody test.

Based on the results of this study, it may be possible to infer the spread of SARS-CoV-2 infection in the region. As already mentioned, HCWs tend to have a high antibody positivity rate; thus, the seroprevalence of anti-SARS-CoV-2 antibodies among residents of Saitama City may be lower than the seroprevalence found in this study.

This study has several limitations. First, as there was only one positive case in the survey, the infection rate in the area was extremely low and the risk of SARS-CoV-2 infection may have been low. As a result of the very low antibody prevalence, the study did not have adequate statistical power to detect epidemiologically meaningful differences in risk factors. Also, statistical analysis with only one positive person may not make sense. The incidence of SARS-CoV-2 infection in Saitama City is likely to remain low; therefore, in order to evaluate the risk factors for SARS-CoV-2 infection in HCWs, it would be useful to continue to follow up the prospective cohort for a long time period or to conduct a case-control study.

Even though the incidence of SARS-CoV-2 infection in Saitama City was low, there were multiple opportunities for medical professionals who were engaged in COVID-19-related medical care to come into contact with patients, and the risk of infection was not negligible. Specifically, none of the 162 HCWs engaged in COVID-19-related care at the hospital were antibody-positive. These results provide evidence that the hospital’s infection control measures to prevent nosocomial infections among HCWs engaging in COVID-19-related care were effective.

Second, participation in the study was limited to those who chose to participate; thus, there is a possibility of selection bias. Some HCWs may have elected not to participate because of anxiety about the possibility of positive results. In the cohort, 93 (84%) participants participated in all three rounds of testing, and the antibody prevalence remained 0%. Even if it is assumed that all those who did not participate in the July round of testing were antibody-positive, there would be no statistically significant difference in the antibody prevalence between HCWs who were and were not engaged in COVID-19 care; thus, participants in the cohort study who did not test in the final round are unlikely to have biased the overall results.

Third, individuals with symptoms of possible infection within the 2 weeks before testing were excluded. Thus, the antibody positivity rate measured may be lower than the true antibody positivity rate in this population. Furthermore, there is a possibility of false-negative results when conducting antibody testing of individuals with asymptomatic infection. Most studies that evaluated the sensitivity of antibody tests were conducted among symptomatic patients, and there is evidence that antibody titers are lower in individuals with asymptomatic infection [11]. In this study, many participants had had no symptoms suggestive of COVID-19 within the previous 4 months. In individuals with asymptomatic infection, the antibody titer may be below the cutoff value. Moreover, the participant who tested positive for antibodies in the study tested negative using the Architect SARS-CoV-2 IgG but positive using the Cica Immuno-test SARS-CoV-2 IgG. The reason for discordant test results using the Architect SARS-CoV-2 IgG is unknown. However, a similar discordance was reported in a study performed in Tokyo [12]. Differences in antibody specificity, measurement methods, cutoff values, and changes in antibody titers over time may have influenced the results.

Finally, this study did not consider the degree of engagement of HCWs in COVID-19-related medical care; thus, the duration of their contact with infected patients is unknown. The risk of infection may vary according to the duration of contact with patients and the specific medical procedure, such as suction of sputum, intubation, and ventilator management. Previous studies have shown that patients with COVID-19 remain infectious for up to 10 days [25]. During this period, HCWs who were directly engaged in patient care should have been classified as “COVID-19-related HCWs;” however, such detailed data were not obtained in this study.

## Conclusion

Of the 607 HCWs in the hospital who were tested for anti-SARS-CoV-2 antibodies, only 1 tested positive, and all staff members engaged in COVID-19-related medical care tested negative. Furthermore, the negative results of the antibody test were maintained over time. These results provide evidence that the infection control measures in the hospital protected HCWs from nosocomial infections.

It is important to conduct regular antibody surveillance and to evaluate the appropriateness of infection control measures, as this enables HCWs to provide care with confidence.

The effectiveness of infection prevention measures at the time of performing medical care procedures such as patient consultation, care, and endoscopic examination should be evaluated prospectively. Furthermore, by conducting antibody tests on residents of Saitama City and comparing the results, it may be possible to identify and quantify risk factors for infection among HCWs. The infection rate in Saitama City is likely to remain low; there is a possibility of an epidemic occurring in the future; therefore, it is necessary to thoroughly prevent infection.

## Supporting information

S1 Table Data set for antibody survey. (XLSX)

S2 Table Data set for hand hygine usage. (XLSX)

S1 Checklist TREND statement checklist. (PDF)

S1 Protocols (PDF)

S2 Protocols (PDF)
