# Supplementary material for: Comprehensive infection control measures prevent hospital-acquired severe acute respiratory syndrome coronavirus 2 infection: A single-center prospective cohort study and seroprevalence survey
Source: PLoS One. 2021 Oct 11;16(10):e0257513. doi: 10.1371/journal.pone.0257513 (PMC8504754; doi:10.1371/journal.pone.0257513)
Supplement: S1 Protocols — (PDF) [file pone.0257513.s004.pdf]

Clinical Research Protocols

1. Name and Category of Research

(1) Title of the Research: Epidemiological Research on SARS-CoV-2 (novel coronavirus) Antibody Positivity Rate among Healthcare Workers at Saitama Medical Center, Jichi Medical University during the COVID-19 Pandemic

(2) Classification of Research

■Research conducted independently by Saitama Medical Center, Jichi Medical University

2, Research implementation

(1) Implementation System in the University

	Affiliation	Title	Name	Roles and Responsibilities	Date of Attendance at the Ethics Seminar	Completion Status of e-learning
1. Principal investigator	Division of General Medicine	Professor	Hitoshi Sugawara	Research Design<br>Research implementation<br>Procurement of reagents<br>Obtaining Consent<br>Specimen collection<br>Data analysis<br>Principal investigator	Attended on October 23, 2018	①April 4, 2020<br>②April 4, 2020
2. Research coordinator	Division of General Medicine	Part-time Physician	Hiroshi Hori	Research Design<br>Research implementation<br>Procurement of reagents<br>Obtaining Consent<br>Specimen collection<br>Data analysis	CREDITS Attended※	Completed
	Division of General Medicine	Associate Professor	Takahiko Fukuchi	Research planning<br>Reagent procurement<br>Data analysis	Attended on January 22, 2019	Completed
	Division of General Medicine	Clinical Assistant Professor	Hanako Yoshihara	Specimen collection<br>Data analysis	CREDITS Attended※	Completed
	Division of General Medicine	Clinical Assistant Professor	Ibuki Kurihara	Specimen collection<br>Data analysis	CREDITS Attended※	Completed
	Division of	Clinical	Ai	Specimen	Attended on	Completed

	General Medicine	Assistant Professor	Kawamura	collection Data analysis	August 22, 2018	
Research Collaborators	Division of Emergency	Professor	Shun Moriya	Implementation of Research	Attended on October 23, 2018	Completed
	Department of Anesthesiology	Professor	Masamitsu Sanui	Implementation of Research	Attended on September 5, 2019.	Completed
	Nursing Department	Manager	Satoko Suzuki	Research Implementation	CREDITS Attended※	Completed

3. Purpose and Significance of the Research

(1) Purpose

- To clarify the prevalence of SARS-COV-2 antibodies among healthcare workers at our center.
- To clarify the characteristics of SARS-COV-2 antibody-positive patients.

(2) Significance

- IgG antibody-positive patients are predicted to have a low risk of reinfection, at least in the short term. This information is particularly useful for health care workers during the COVID-19 pandemic in terms of nosocomial infection prevention.

4. Research Methods and Duration

Research Methods

Research Design

■Clinical Research

■Observational research

Specific Outline

- The subject shall be from volunteers recruited by posting posters in the Research Implementation Section and Research Cooperation Section. An email shall be sent to staff members who have expressed interest in volunteering, and if they respond to the email, they shall be considered to be willing to participate.
- The researcher shall explain the research directly to the volunteers who responded to the email and give them the explanation and consent forms.
- At a later date, the volunteer who signed the consent form shall be the research subject (doctor or nurse).
- Blood samples shall be collected from all the subjects at the start of the research, one month after the start of the research, and two months after the start of the research, and the serum shall be frozen and stored. Two to four weeks after the start of the research, each of the stored specimens at the start of the research shall be subjected to a qualitative test for SARS-CoV-2 IgG/IgM antibodies by immunochromatography. Three months after the start of the research, the same qualitative test shall be performed on each of the remaining specimens.
- A research reagent, (the Novel Coronavirus Antibody Test Kit (COVID-19 IgG/IgM Immunochromatographic Kit, Innovita (Tangshan) Biological Technology Co.) shall be used for antibody testing. For judgment, it shall be confirmed that the control is positive, check the IgG band, and classify the result as "IgG antibody positive," "IgG antibody positive," or "unable to judge. The test results should also be stored in a photograph.

- This research is to determine the history of infection. Therefore, IgM shall not be measured; if a kit with IgM results is used, it shall be labeled so that the results are not visible.
- The results should be visually checked by the Principal Investigator and one of the Research Coordinators, and any matches should be recorded. If the results are inconsistent, a third Research Coordinator shall make the determination.
- This test kit is a research reagent only and it is clearly labeled as "may not be used for therapeutic or diagnostic purposes".
- The percentage of antibody-positive subjects among all the subjects shall be measured.
- Following items as at the time of the research shall be recorded: The subject's age, gender, history of living abroad, city or town of residence, underlying medical conditions, medication history, whether or not they have received medical checkups, smoking history, alcohol consumption history, place of residence within the past year, department of work, BCG intake history, the presence and timing of COVID19 (novel coronavirus infection) in patients under their care (location, date and time), symptoms of the common cold (cough, nasal discharge, sore throat, malaise, taste disorder, respiratory distress) within the last 3 months and the date (how many days before the examination), and the presence of fever.
- Analyze whether there are statistically significant differences in the proportions of these factors among the subjects who test positive for antibodies compared to the subjects who test negative.
- All the subjects shall be asked every month about the presence or absence of airway symptoms, fever, taste disturbance, and COVID-19 diagnosis at a medical institution in the last month. This shall be continued for the duration of the research.
- As of April 6, 2020, there is no antibody test that can guarantee high accuracy. Research on antibody accuracy verification is ongoing worldwide, and it is possible that antibody test kits with verified/guaranteed accuracy will be identified in the future. If antibody tests with proven accuracy are available during the research period, the research reagents may be changed.

Expected Number of Research Subjects and Rationale for Setting

Planned number of research subjects: 60

Rationale: Looking at the increasing tendency of infection, it is predicted that the IgG antibody positive rate as of April 2020 is quite low, but in Japan, there are no statistics so far and it is difficult to make an accurate assumption. The sample size would be 35 or less with a margin of error of 10% and a confidence level of 95%, and 49 or less with 15% or less under the same conditions. The total number of participants is set at 60, including 10 who withdrew during the course of the research. If the number of positive subjects is less than 5% at the beginning of the research, additional recruitment may be conducted.

Method of Statistical Analysis, Evaluation Items and Methods

a. Method of Statistical Analysis

- The actual number of subjects who tested positive for antibodies at the beginning of the research, after 1 month, and after 2 months, and the percentage of all subjects is measured, and 95% confidence intervals is obtained.
- Obtain the aforementioned predetermined factors: The subject's age, gender, history of living abroad, city of residence, underlying illness, medication history, whether or not he or she has received medical checkups, smoking history, drinking history, place of residence within the past year, department of work, BCG intake history, the presence or absence of COVID-19 (novel coronavirus infection) in the patient in charge, history of travel in the last year, the presence or absence of access to cluster infection sites, the presence or absence of the common cold symptoms (cough, nasal discharge, sore throat, malaise, dysgeusia, respiratory distress) within 3 months, and the presence or absence of fever.

The differences in these factors between IgG antibody-positive and antibody-negative subjects are tested by chi-square test, Fisher's exact test, and logistic analysis, and for continuous variables by Mann-Whitney U test and logistic analysis. In addition, the characteristics of the above factors in the research subjects who test positive for antibodies will be described.

- Measure the percentage of antibody-positive individuals who develop COVID-19 after a positive test.

b. Evaluation Items

- The primary outcome is the rate of antibody positivity in the entire research population, and the change in the rate of positivity
- The secondary outcome is the detection of significant differences in each factor among antibody-positive and antibody-negative subjects (age, sex, place of residence, department, the presence of COVID19 in the patient in charge if not an early resident, travel history in the past year, access to cluster-infected areas, the presence of cold symptoms and fever within 3 months), and subsequent incidence of COVID-19 in antibody-positive individuals.

Research Period

- The research shall start within one month after the approval.
- The research period shall be 4 months. It may be postponed for up to one year.
- To be published in a conference or paper within one year after completion of the analysis.

5. Selection of Research Subjects

(1) Number of Subjects, etc.

1) Number and Type of Subjects

■Healthy people (approx. 60 or more people [of which approx. 60 are healthy people in the University])

2) Target age range

■ Limited (20 years or older)

3) Gender

■ Both

4) Recruitment Method

■ Poster

(Posters are posted in Division of General Medicine, Emergency Center Medical Office and Nursing Station, Intensive Care Unit Medical Office and Nursing Station, Resident's Office, Ward 2A Nursing Station)

■ Others (Volunteers are recruited and Email is sent back to the applicants)

(2) Selection Policy

Selection criteria: Healthy medical personnel who are engaged in this hospital including junior resident, physicians in general medicine Division, physicians in emergency department, physicians ICU, EICU nurses, emergency and ward 2A nurses who have given consent for this research, who have been or will be engaged in the treatment of SARS-COV-2 infected patients

Exclusion criteria: Those with fever, cough, or respiratory distress at the time of the initial examination, and those who do not consent to the research.

6. Scientific Rationality

- Currently, PCR tests for SARS-CoV-2 infection are less common in Japan than in other countries, and the actual spread of the infection is not known. By detecting antibody positivity, the percentage of previously infected individuals in the control group can be measured.

- Since it is currently difficult to assume the SARS-CoV-2 infection rate, the followings are the subject of the research: Physicians in general medicine and emergency departments who have frequent contact with patients diagnosed with COVID-19 or with patients who complain of respiratory tract symptoms such as the common cold, physicians in ICU, ward and emergency nurses who have actually been involved in the treatment of COVID-19, and junior resident as healthy adults who rotate through multiple departments.
- For statistical analysis, all antibody-positive subjects are assumed to have a history of infection, and the percentage of subjects tested is determined.

7. Procedures for Obtaining Informed Consent, etc.

(1) Collection of Samples and Information, etc.

■Use of new samples and information

The content: The COVID-19 IgG/IgM immunochromatography kit (sold by TK Research Co., Ltd.) is used, and the stored serum of the research subjects is used. Have them fill out a research statement, consent form, and a separate questionnaire for each research subject.

■Obtain consent in writing

(2) Selection of a Substitute

■No Substitute

■This is not research using a substitute (including cases where opt-out by the substitute is allowed)

(3) Procedures for Obtaining Informed Assent (oral assent is generally for minors of elementary school age and older but less than 16 years of age; written assent is generally for minors of junior high school age and older but less than 16 years of age)

■Do not obtain informed assent

■There is no need to obtain informed assent.

8. Handling of Personal Information, etc.

(1) Collection of samples, information, etc.

1) Samples (blood, human tissue, etc.)

■Samples collected from: ■Healthy individuals

■Number of times blood is collected: (3) times during the period

Amount per time: (about 5) ml

Method of collection

■To be collected only for the purpose of conducting this research (not related to medical treatment or therapy)

2) Information (medical information, questionnaire survey, etc.)

■Collect information

■Questionnaires, etc.

(2) Anonymization of Samples and Information, etc.

■Anonymize samples and information

Anonymization (with the correspondence table)

Time of anonymization: ■A fixed time during the research period

Method of anonymization: Create an ID (serial number) for each research subject. Create a correspondence chart that records the name, staff ID, and contact information for each ID. Upon obtaining the test results, record the test results for each subject ID. Only Hiroshi Hori has access to the correspondence table of anonymization.

Reasons for creating a correspondence table:

■The data may be revised, changed, or added as necessary to ensure the accuracy, scientificity, and quality of the research after anonymization, and should be traceable.

■It is necessary to identify the relevant data when a subject withdraws consent or refuses to participate in the research.

(3) Joint use of samples and information, etc.

■There is no exchange of samples and information with collaborating institutions.

9. Comprehensive Assessment of Burdens and Anticipated Risks and Benefits to Research Subjects, Measures to Minimize Such Burdens and Risks

(1) Burden Incurred by Research Subjects

1) Cost burden

There is no cost burden on research subjects when they participate in the research.

2) Other burdens

There is no other burden on research subjects when they participate in the research.

(2) Anticipated Risks to Research Subjects

1) Disadvantage ■ None

2) Risks ■None

3) Discomfort ■Yes Description: Pain associated with blood collection

(3) Benefits Accruing to the Research Subjects

1) Gratuities to subjects

■None

2) Other benefits

■No

(4) A comprehensive Assessment of the Burdens and Anticipated Risks and Benefits to Research Subjects, and Measures to Minimize Such Burdens and Risks

1) Overall evaluation

- The possibility of direct disadvantage or benefit to the research subjects is low.

2) Measures to minimize burden and risk

Minimize pain during blood collection.

- The pain during the blood collection process should be minimized
- The test should be performed during or after work breaks to minimize the possibility that work may be interfered
- There is a possibility of interference with work due to infectivity evaluation (PCR test, etc.) in the case of antibody positivity. Therefore, by allowing a period of one month from the time of blood collection to the time of antibody measurement, the infectivity evaluation shall be unnecessary and the burden shall be eliminated.
- In order to minimize the psychological burden of knowing the results, the results shall be disclosed only to those who wish to receive them.

3) Compensation for loss

There shall be no compensation for losses incurred through the implementation of this research.

10. Storage of Samples and Information under Research, Storage and Disposal of Records Relating to the Transfer of Samples and Information

(1) Samples and Information, etc., and Retention of Samples and Information during Research

1) Type of samples and information

■Original samples and source materials (case reports, survey sheets, etc.)

■Processed materials

■Consent form
■Anonymization correspondence table

2) Form of samples and information, etc.

■Information in paper form
■Electronic information
■Samples

3) Retention location

■Other (Specific location: Lockable cabinet in a doctor's office on the 5th floor of the Administration and Research Building, Saitama Medical Center, Jichi Medical University)

(2) Relating of Samples and Information, etc. after Completion of Research

■After the research is completed, the samples and information shall be stored for use for purposes other than the research purpose of this application.

Type of samples/information, etc.: Materials of test results, anonymization correspondence table

Reason for retention: To be used as reference material for future clinical trials

■When using stored samples and information for other purposes, apply to the Ethics Committee again and obtain approval.

■When using stored samples and information for other purposes, obtain the subject's consent again.

Retention location

■Other (Specific location: Lockable cabinet in a doctor's office on the 5th floor of the Administration and Research Building, Saitama Medical Center, Jichi Medical University)

(3) Method of destruction and disposal of samples and information, etc.

■Use a shredder to cut them.
■Others (USB memory and PC data should be erased using erasure software, and blood samples should be destroyed)

11. Content and Method of Reporting to the President (except for reporting of serious adverse events)

■Once a year, the progress of the clinical research and the occurrence of adverse events and problems shall be reported to the President of the University without delay by means of Progress Report on Clinical Research.

■When the clinical research is discontinued, it shall be promptly reported to the President in the form of Clinical Research Discontinuation Report.

■When the clinical research is terminated, it shall be promptly reported to the President in the form of Clinical Research Termination Report.

12. Sources of Research Funding, Conflict of Interest Related to Research and Personal Earnings, Status of Conflict of Interest Related to Research by Researchers, etc.

(1) Funding Sources

■Other: Scholarship donations

(2) Relationship of the researchers and other related organizations

・The Principal Investigator is a physician in the department, and the Research Coordinators are full-time or part-time physicians in the Division of General Medicine of the Hospital.

(3) Conflict of interest

There are no conflicts of interest to be reported in this research.

(4) Benefits to be gained from the research

■None

(5) Patent rights, etc.

■There is no possibility that patent rights, etc. will arise.

13. How to Disclose Information on Research

(1) Registration of Research Summary and Results

■Register the summary and results of the research.

Where to register ■University hospital Medical Information Network (UMIN)

(2) Disclosure of personal data (test results, etc.) newly obtained in the research

■There is personal data to be newly obtained in this research.

■Newly obtained personal data will be disclosed to the individual.

■Disclosure to those who wish to receive it

(3) Publication of research results

■Publication of research results

■Method of publication: ■Publication

14. Handling of Research in a Situation Where Research Subjects are in Immediate and Obvious Danger of Death

■The research is not conducted in a situation where the life of the research subject is in immediate and obvious danger.

15. Responses to the Event of a Serious Adverse Event

■ This research does not involve invasion (there is no invasion or there is only minor invasion) [Research that is exempted from reporting].

16. Existence of Compensation for Health Damage and its Details

■ This research does not involve invasion (there is no invasion or there is only minor invasion) [Research not covered by compensation].

17. Responses to the Provision of Medical Care after the Research is Conducted

■This is research that does not exceed normal medical treatment or does not involve medical treatment

18. Handling of Research Results Pertaining to Research Subjects

■There is no possibility of gaining important insights into the health of research subjects or genetic characteristics that can be passed on to offspring.

19. Outsourcing of Research-Related Tasks

■No outsourcing of research-related tasks

20. Monitoring and Auditing System and Implementation Procedures

Monitoring and auditing will not be conducted [Exempted research].

21. Inquiries about Research and Responses to Complaints

(1) Contact

Affiliation: Saitama Medical Center, Jichi Medical University

Title : Professor Name : Hitoshi Sugawara
Phone Number: 048-647-2111
Campus Extension: None PHS (if available): 5565
E-mail : hsmdfacp@jichi.ac.jp

(2) Contact for Complaints

Division of General Medicine, Saitama Medical Center, Jichi Medical University
(Phone: 048-648-5225)
