# Supplementary material for: Comprehensive infection control measures prevent hospital-acquired severe acute respiratory syndrome coronavirus 2 infection: A single-center prospective cohort study and seroprevalence survey
Source: PLoS One. 2021 Oct 11;16(10):e0257513. doi: 10.1371/journal.pone.0257513 (PMC8504754; doi:10.1371/journal.pone.0257513)
Supplement: S2 Protocols — (PDF) [file pone.0257513.s005.pdf]

Clinical Research Protocols

1. Name and Category of Research

(1) Title of the Research: Epidemiological Research on SARS-CoV-2 (novel coronavirus) Antibody Positivity Rate among Healthcare Workers at Saitama Medical Center, Jichi Medical University

(2) Classification of Research

■Research conducted independently by Saitama Medical Center, Jichi Medical University

2. Research Implementation Structure

(1) Implementation System in the University

	Affiliation	Title	Name	Roles and Responsibilities	Date of Attendance at the Ethics Seminar	Completion Status of e-learning
1. Principal investigator	Division of General Medicine	Professor	Hitoshi Sugawara	Research Design<br>Research implementation<br>Procurement of reagents<br>Obtaining Consent<br>Specimen collection<br>Data analysis<br>Principal investigator	Attended on October 23, 2018	①April 4, 2020<br>②April 4, 2020
2. Research coordinator	Division of General Medicine	Part-time Physician	Hiroshi Hori	Research Design<br>Research implementation<br>Procurement of reagents<br>Obtaining Consent<br>Specimen collection<br>Data analysis	CREDITS Attended※	Completed
	Division of General Medicine	Associate Professor	Takahiko Fukuchi	Research planning<br>Reagent procurement<br>Data analysis	Attended on January 22, 2019	Completed
	Division of General Medicine	Clinical Assistant Professor	Hanako Yoshihara	Specimen collection<br>Data analysis	CREDITS Attended※	Completed
	Division of General Medicine	Clinical Assistant Professor	Ibuki Kurihara	Specimen collection<br>Data analysis	CREDITS Attended※	Completed
Research Collaborators	Department of	Professor, Center	Shunsuke Endo	Research Design<br>Research	CREDITS Attended※	Completed

	Respirator Surgery	Director		Implementation		
	Nursing Department	Manager	Satoko Suzuki	Research Implementation	CREDITS Attended※	Completed

3. Purpose and Significance of the Research

(1) Purpose

- To verify the effectiveness of infection control measures by clarifying the prevalence of SARS-COV-2 antibodies among healthcare workers at our center.
- To clarify the characteristics of SARS-COV-2 antibody-positive healthcare workers at our center.

(2) Significance

- Prevention of infection among health care workers is very important since most of the infection-related deaths in Japan were due to healthcare-associated infections.
- Our center treated patients with severe COVID-19 and some patients with moderate disease. In the emergency cases, our center thoroughly has taken measures to prevent infection by assigning inpatient beds based on the COVID-19 prediction score, isolating and zoning infected patients, assigning full-time medical personnel to dedicated wards, and implementing thorough ventilation and social distance in the hospital in order for the infection prevention measures to be implemented.
- By comparing the antibody-positive rate in patients with and without COVID-19 treatment, and by comparing the results of other studies of antibody-positive rates in healthy individuals in the city, we evaluate the risk of infection in medical treatment and how effective the infection prevention measures are.
- The antibody positivity rate of SARS-COV-2 in our center can be an indicator to estimate the infection status in Saitama City area.

4. Research Methods and Duration

(1) Research Methods

1) Research Design

a. ■Clinical Research

■Observational research

■No invasion or minor invasion

Specific Outline

- The subjects shall be medical personnel (physicians, nurses, nursing assistants, nursing aides, therapists, pharmacists, and radiologists) working at the Center.
- Volunteers shall be recruited through the posters (with application forms hanging from them) posted in the Research Implementation Department and Research Cooperation Department and by sending them to the hospital's e-mail address. Only when we receive an application form, email, or phone call from an individual who wishes to participate, we assume that they are willing to participate.
- The researcher shall provide a written explanation of the research to the volunteers who have expressed their willingness to participate, and give them an explanation form and a consent form.
- Volunteers who sign the consent form shall be considered research subjects.
- Blood samples shall be collected from all research subjects and labeled with an anonymized ID. The specimens shall be collected by SRL, a private laboratory, and SRL shall be informed of the anonymized ID only and shall not be provided with the subject's personal information. The handling of the specimen as a biosafety level shall be in accordance with SRL policy.
- Two weeks after the start of the research, the laboratory shall send us the results of the antibody assay

in writing.

- The antibody to be used shall be Roche's Elecsys Anti-SARS-CoV-2.
- This antibody is available for purchase in Japan and has been approved for emergency use by the FDA. This antibody is highly accurate with a sensitivity of 100% (95% CI: 88.1-100%) and specificity of 99.81% (95% CI: 99.65-99.91%).
- Elecsys Anti-SARS-CoV-2 (Roche) is measured by CLEIA method and classified as "antibody positive", "antibody negative", or "unable to judge". Along with these results, the measured values are also recorded. These results shall be recorded as soon as the results are obtained from private laboratories.
- The percentage of antibody-positive subjects among all the subjects shall be measured.
- Following items as at the time of the research shall be recorded in the questionnaire: The subject's age, sex, city of residence, the presence or absence of underlying illness, smoking history, department of employment, history of BCG intake, the presence or absence of COVID19 (novel coronavirus infection) in the patient and its timing, travel history in the last year, the presence or absence of access to cluster-infected areas (location, date and time), the presence or absence of the common cold symptoms (cough, nasal discharge, sore throat, malaise, dysgeusia, fever) within 4 months and its date (how many days before the test), the presence or absence of fever and date (days before the test), and the presence of fever
- Analyze whether there are statistically significant differences in the proportions of these factors among the subjects who test positive for antibodies compared to the subjects who test negative.
- The research reagents may be changed if antibody tests with proven higher accuracy are available during the research period.

Expected Number of Research Subjects and Rationale for Setting

Planned number of research subjects: 400 or more

Rationale: There are limited studies of healthcare professionals in Japan and no data in Saitama Prefecture as of June 4, 2020. Since the positivity rate was less than 1% in our research of medical personnel engaged in COVID-19 treatment, 381 subjects are the required sample size with a predicted positivity rate of 1%, a margin of error of 1%, and a confidence level of 95%. Therefore, the recruitment size should be 400 or more.

Method of Statistical Analysis, Evaluation Items and Methods

a. Method of Statistical Analysis

- The actual number of subjects who tested positive for antibodies, as a percentage of all subjects, is measured and a 95% confidence interval is obtained.
- Obtain the aforementioned predetermined factors: Age, sex, city of residence, the presence or absence of underlying illness, smoking history, department of employment, history of BCG intake, the presence or absence of COVID19 (novel coronavirus infection) in the patient and its timing, travel history in the last year, the presence or absence of access to cluster-infected areas (location, date and time), the presence or absence of the common cold symptoms (cough, nasal discharge, sore throat, malaise, dysgeusia, fever) within 4 months and its date (how many days before the test), the presence or absence of fever and date (days before the test), and the presence of fever. Test for differences in these factors between antibody-positive and antibody-negative subjects by chi-square test, Fisher's exact test, and logistic analysis, and for continuous variables by Mann-Whitney U test and logistic analysis. Further, describe the characteristics of the above factors in the research subjects who test positive for antibodies.

b. Evaluation Items

- The primary outcome is the difference between the antibody-positive rate among all research subjects

and the rate among COVID-19 practitioners.

- The secondary outcome is the detection of significant differences in each factor between antibody-positive and antibody-negative subjects (Age, gender, place of residence, department, presence of COVID19 in the patient in charge, travel history in the last year, access to cluster-infected areas, the common cold symptoms and fever within 4 months).
- Data from health care providers in previous studies shall also be evaluated.

Research Period

- The research shall start within one month after the approval.
- The research period shall be 3 months.
- To be published in a conference or paper within one year after completion of the analysis.

5. Selection of Research Subjects

(1) Number of Subjects, etc.

1) Number and Type of Subjects

■ Healthy people (approx. 400 or more people [of which approx. 400 are healthy people in the University])

2) Target age range

■ Limited (20 years or older)

3) Gender

■ Both

4) Recruitment Method

■ Poster

(Posters are posted in each ward (3A, 5A, 5B, 6A, 6B, 4East, 4West, 5East, 5West, 6East, 6West nursing stations, medical office on the 3rd floor of the South Building, outpatient treatment room, emergency room, emergency ward, EICU staff room, ICU staff room), Pharmacy Department, Rehabilitation Department Staff Room, Nursing Assistant Staff Room, Radiology Technician Room)

■ Others (Volunteer recruitment via Email: The Center's staff of physicians, nurses, pharmacists, nursing assistants, therapists, and radiologists shall be notified by e-mail. The in-hospital email or the one registered as @ jichi.ac.jp shall be used.

(2) Selection Policy

Selection criteria: Healthy medical personnel who are engaged in this hospital and who have given consent to this research.

Exclusion criteria: Those with symptoms of COVID-19 within 2 weeks of the test, and those who do not consent to the research.

6. Scientific Rationality

- At present, the number of patients infected with SARS-CoV-2 is lower in Japan than in other countries, and the actual situation of the spread of infection is not known. It is important to estimate the degree of existing infection in the region since the degree of prevalence varies from region to region. Furthermore, prevention of infection among health care workers is very important since many infection-related deaths were due to healthcare-associated infections. Therefore, this research can be a means of verifying how effective the contact and droplet prevention measures are in preventing infections in medical institutions engaged in COVID-19 treatment.
- For statistical analysis, all antibody-positive subjects are assumed to have a history of infection, and the percentage of subjects tested is determined.

7. Procedures for Obtaining Informed Consent, etc.

(1) Collection of Samples and Information, etc.

■Use of new samples and information

The content: Elecsys Anti-SARS-CoV-2 (Roche) will be used.
The serum of the research subjects will be used.
Have the subjects complete a research statement, consent form, and a separate medical questionnaire for each research subject.

■Obtain consent in writing

8. Handling of Personal Information, etc.

(1) Collection of samples, information, etc.

1) Samples (blood, human tissue, etc.)

■Samples collected from: ■Healthy individuals

■Number of times blood is collected: (1) times during the period
Amount per time: (about 5) ml

Method of collection

■To be collected only for the purpose of conducting this research (not related to medical treatment or therapy)

2) Information (medical information, questionnaire survey, etc.)

■Collect information

■Questionnaires, etc.

(2) Anonymization of Samples and Information, etc.

■Anonymize samples and information

Anonymization (with the correspondence table)

Time of anonymization: ■A fixed time during the research period

Method of anonymization: Create an ID (serial number) for each research subject. Create a correspondence chart that records the name, staff ID, and contact information for each ID. Upon obtaining the test results, record the test results for each subject ID. Only Hiroshi Hori has access to the correspondence table of anonymization.

Reasons for creating a correspondence table:

■The data may be revised, changed, or added as necessary to ensure the accuracy, scientificity, and quality of the research after anonymization, and should be traceable.

■It is necessary to identify the relevant data when a subject withdraws consent or refuses to participate in the research.

(3) Joint use of samples and information, etc.

■There is no exchange of samples and information with collaborating institutions.

9. Comprehensive Assessment of Burdens and Anticipated Risks and Benefits to Research Subjects, Measures to Minimize Such Burdens and Risks

(1) Burden Incurred by Research Subjects

1) Cost burden

There is no cost burden on research subjects when they participate in the research.

2) Other burdens

There is no other burden on research subjects when they participate in the research.

(2) Anticipated Risks to Research Subjects

1) Disadvantage ■ None

2) Risks ■None

3) Discomfort ■Yes Description: Pain associated with blood collection

(3) Benefits Accruing to the Research Subjects

1) Gratuities to subjects

■None

2) Other benefits

■No

(4) A comprehensive Assessment of the Burdens and Anticipated Risks and Benefits to Research Subjects, and Measures to Minimize Such Burdens and Risks

1) Overall evaluation

- The possibility of direct disadvantage or benefit to the research subjects is low.

2) Measures to minimize burden and risk

Minimize pain during blood collection.

- The pain during the blood collection process should be minimized
- The test should be performed during or after work breaks to minimize the possibility that work may be interfered
- There is a possibility of interference with work due to infectivity evaluation (PCR test, etc.) in the case of antibody positivity. Therefore, by allowing a period of one month from the time of blood collection to the time of antibody measurement, the infectivity evaluation shall be unnecessary and the burden shall be eliminated.
- In order to minimize the psychological burden of knowing the results, the results shall be disclosed only to those who wish to receive them.

3) Compensation for loss

There shall be no compensation for losses incurred through the implementation of this research.

10. Storage of Samples and Information under Research, Storage and Disposal of Records Relating to the Transfer of Samples and Information

(1) Samples and Information, etc., and Retention of Samples and Information during Research

1) Type of samples and information

■Original samples and source materials (case reports, survey sheets, etc.)

■Processed materials

■Consent form

■Anonymization correspondence table

2) Form of samples and information, etc.

■Information in paper form

■Electronic information

■Samples

3) Retention location

■Other (Specific location: Lockable cabinet in a doctor's office on the 5th floor of the Administration and Research Building, Saitama Medical Center, Jichi Medical University)

(2) Relating of Samples and Information, etc. after Completion of Research

■After the research is completed, the samples and information shall be stored for use for purposes other than the research purpose of this application.

Type of samples/information, etc.: Materials of test results, anonymization correspondence table

Reason for retention: To be used as reference material for future clinical trials

■When using stored samples and information for other purposes, apply to the Ethics Committee again and obtain approval.

■When using stored samples and information for other purposes, obtain the subject's consent again.

Retention location

■Other (Specific location: Lockable cabinet in a doctor's office on the 5th floor of the Administration and Research Building, Saitama Medical Center, Jichi Medical University)

(3) Method of destruction and disposal of samples and information, etc.

■Use a shredder to cut them.

■Others (USB memory and PC data should be erased using erasure software, and blood samples should be destroyed)

11. Content and Method of Reporting to the President (except for reporting of serious adverse events)

■Once a year, the progress of the clinical research and the occurrence of adverse events and problems shall be reported to the President of the University without delay by means of Progress Report on Clinical Research.

■When the clinical research is discontinued, it shall be promptly reported to the President in the form of Clinical Research Discontinuation Report.

■When the clinical research is terminated, it shall be promptly reported to the President in the form of Clinical Research Termination Report.

12. Sources of Research Funding, Conflict of Interest Related to Research and Personal Earnings, Status of Conflict of Interest Related to Research by Researchers, etc.

(1) Funding Sources

■Other: Scholarship donations

(2) Relationship of the researchers and other related organizations

・The Principal Investigator is a physician in the department, and the Research Coordinators are full-time or part-time physicians in the Division of General Medicine of the Hospital.

(3) Conflict of interest

There are no conflicts of interest to be reported in this research.

(4) Benefits to be gained from the research

■None

(5) Patent rights, etc.

■There is no possibility that patent rights, etc. will arise.

13. How to Disclose Information on Research

(1) Registration of Research Summary and Results

■Register the summary and results of the research.

Where to register ■University hospital Medical Information Network (UMIN)

(2) Disclosure of personal data (test results, etc.) newly obtained in the research

■There is personal data to be newly obtained in this research.

■Newly obtained personal data will be disclosed to the individual.

■Disclosure to those who wish to receive it

(3) Publication of research results

■Publication of research results

■Method of publication: ■Publication

14. Handling of Research in a Situation Where Research Subjects are in Immediate and Obvious Danger of Death

■The research is not conducted in a situation where the life of the research subject is in immediate and obvious danger.

15. Responses to the Event of a Serious Adverse Event

■ This research does not involve invasion (there is no invasion or there is only minor invasion) [Research that is exempted from reporting].

16. Existence of Compensation for Health Damage and its Details

■ This research does not involve invasion (there is no invasion or there is only minor invasion) [Research not covered by compensation].

17. Responses to the Provision of Medical Care after the Research is Conducted

■This is research that does not exceed normal medical treatment or does not involve medical treatment

18. Handling of Research Results Pertaining to Research Subjects

■There is no possibility of gaining important insights into the health of research subjects or genetic characteristics that can be passed on to offspring.

19. Outsourcing of Research-Related Tasks

■Outsourcing research-related tasks

■Contracts available (planned)

Details of contract work: Antibody testing at SRL

Method of supervision of contractors: No personal information shall be provided

20. Monitoring and Auditing System and Implementation Procedures

Monitoring and auditing will not be conducted [Exempted research].

21. Inquiries about Research and Responses to Complaints

(1) Contact

Affiliation: Saitama Medical Center, Jichi Medical University
Title : Professor Name : Hitoshi Sugawara
Phone Number: 048-647-2111
Campus Extension: None PHS (if available): 5565
E-mail : hsmdfacp@jichi.ac.jp

(2) Contact for Complaints

Division of General Medicine, Saitama Medical Center, Jichi Medical University
(Phone: 048-648-5225)
